# HDL surface lipids mediate CETP binding as revealed by electron microscopy and molecular dynamics simulation

**DOI:** 10.1038/srep08741

**Published:** 2015-03-04

**Authors:** Meng Zhang, River Charles, Huimin Tong, Lei Zhang, Mili Patel, Francis Wang, Matthew J. Rames, Amy Ren, Kerry-Anne Rye, Xiayang Qiu, Douglas G. Johns, M. Arthur Charles, Gang Ren

**Affiliations:** 1 The Molecular Foundry, Lawrence Berkeley National Laboratory, Berkeley, CA 94720; 2 Centre for Vascular Research, University of New South Wales, Kensington, Sydney, NSW 2052, Australia; 3 Pfizer Inc., Groton, Connecticut 06340, USA; 4 Merck Research Laboratories, Rahway, New Jersey 07065, USA; 5 School of Medicine, University of California, San Francisco, California 94115, USA

## Abstract

Cholesteryl ester transfer protein (CETP) mediates the transfer of cholesterol esters (CE) from atheroprotective high-density lipoproteins (HDL) to atherogenic low-density lipoproteins (LDL). CETP inhibition has been regarded as a promising strategy for increasing HDL levels and subsequently reducing the risk of cardiovascular diseases (CVD). Although the crystal structure of CETP is known, little is known regarding how CETP binds to HDL. Here, we investigated how various HDL-like particles interact with CETP by electron microscopy and molecular dynamics simulations. Results showed that CETP binds to HDL via hydrophobic interactions rather than protein-protein interactions. The HDL surface lipid curvature generates a hydrophobic environment, leading to CETP hydrophobic distal end interaction. This interaction is independent of other HDL components, such as apolipoproteins, cholesteryl esters and triglycerides. Thus, disrupting these hydrophobic interactions could be a new therapeutic strategy for attenuating the interaction of CETP with HDL.

An elevated level of low-density lipoprotein-cholesterol (LDL-C) and/or a low level of high-density lipoprotein cholesterol (HDL-C) in human plasma are major risk factors for cardiovascular disease (CVD). Cholesteryl ester transfer protein (CETP), with a molecular mass of 53 kDa (composed of 476 amino acids) before post-translational modification^1^, mediates the cholesteryl ester (CE) transfer from high-density lipoprotein (HDL) to low-density lipoprotein (LDL) and very low-density lipoprotein (VLDL), and in exchange for triglyceride (TG). CETP deficiency has been shown to be associated with elevated HDL cholesterol levels^2^,^3^. Loss-of-function mutations in the CETP gene were negative associated with premature atherosclerosis, and have been implicated in longevity in some studies^2^. On the other hand, there is also evidence that CETP mutations are associated with an increased incidence of coronary heart diseases^4^. Despite these inconsistencies and because of the urgent public desire to expand treatment options beyond statins, the most financially successful drug to reduce LDL-C levels to date, CETP has been used as a promising drug target for designing inhibitors in order to treat heart disease^5^,^6^,^7^,^8^,^9^,^10^. Four large clinical trials of CETP inhibitors^5^,^8^,^9^,^10^ have been undertaken to date. The first two CETP inhibitors failed in phase III clinical trials due to increase mortality related to off-target effects and lack of efficacy. Two other CETP inhibitors^9^,^10^ are currently being investigated in large clinical outcome trials. As such, an improved understanding of CETP's molecular interactions could eventually provide beneficial and definitive descriptions of CETP function, thereby leading to new CETP-related drug design.

The structure of CETP, revealed by X-ray crystallography, resembles a banana shape with dimensions of roughly 3 × 3 × 13 nm and contains four structural components: an N-terminal β-barrel domain, a C-terminal β-barrel domain, a central β-sheet, and a C-terminal extension (a distorted amphipathic helix, helix X, Glu465-Ser476 at the C terminus)^11^,^12^. Electron microscopy (EM) has shown that CETP forms a bridge between HDL and LDL, with its N-terminal β-barrel domain penetrating the HDL surface and its C-terminal β-barrel domain penetrating the LDL surface^13^. A molecular dynamics (MD) simulation has revealed that the distal portions of the N- and C-terminal β-barrel domains of CETP remain highly flexible in solution^14^. This flexibility may be necessary for conformational changes to occur at the distal ends, a necessary step for the formation of a tunnel through the entire molecule^13^,^15^.

Although CETP has been intensively studied, a detailed understanding of how CETP senses and binds to HDL remains unknown due to the heterogeneity and dynamics of HDL. HDLs vary in size, shape, and composition^16^,^17^. Considering that most plasma CETPs are naturally bound to HDLs^18^, an investigation of how different components of HDL affect the interaction of spherical HDL with CETP is essential for a complete understanding of CETP function.

Here, we studied CETP interactions with various HDLs and liposome vesicles using optimized negative-staining (OpNS), cryo-electron tomography (Cryo-ET), and molecular dynamics simulations in order to understand how the different HDL components affect CETP binding.

## Results

### EM images of CETP bound to plasma HDL_2_

Based on previous reports, the spherical HDL_2_ in plasma varies in diameter (~9 to ~15 nm) and in density (from 1.063 to 1.125 g/ml)^19^,^20^. Earlier studies^18^,^19^,^21^ showed the average molecular weight of HDL_2_ is 360 kDa and that the particles contain three major surface components: apolipoprotein A-I and A-II (apoA-I and apoA-II) (total ~40.2% of MW) and phospholipids (~31.3% of MW), as well as core lipids: cholesterol esters (~17.6% of MW) and triglycerides (~4.2% of MW). A small amount of free cholesterol (~5.8% of MW) is distributed between the particle surface and the core^21^.

**Figure 1:**
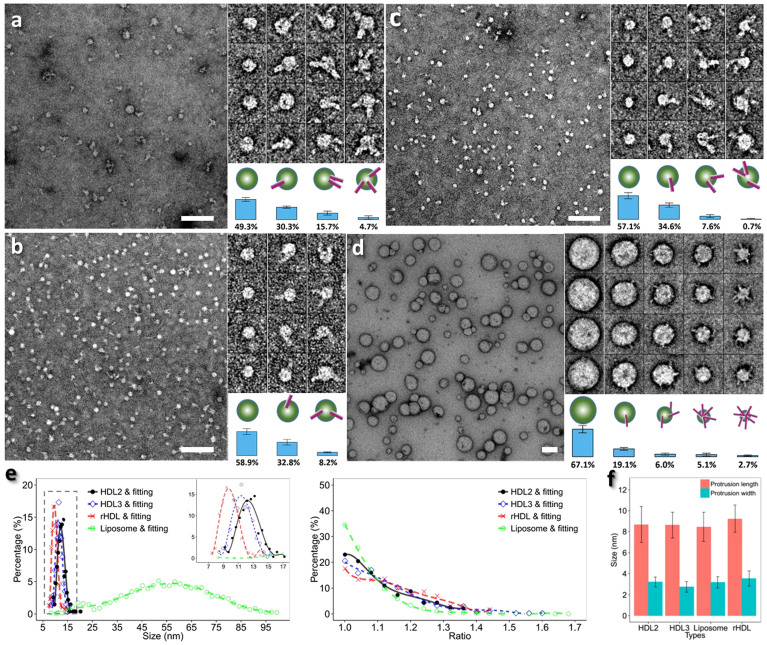
Negative-staining electron micrographs of HDL subclasses and liposome incubation with CETP by optimized negative-staining (OpNS). The samples of CETP incubated with, (a) plasma HDL_2_, (b) plasma HDL_3_, (c) reconstituted spherical HDL (rHDL), and (d) 1-Palmitoyl-2-oleoylphosphatidylcholine (POPC) liposome vesicles were prepared by optimized negative-staining (OpNS). Survey view (left panel), representative conformations of particles (top right panel), and schematics of the particle structures and percentages of each conformation (bottom right panels). (e) Histogram of particle diameter (left panel, sampling step 0.5 nm, fitted with sixth-degree polynomial functions) and shape (right panel, sampling step 0.04, fitted with sixth-degree polynomial functions). The diameter is calculated based on the geometric mean of two measured diameters (in which one is the longest direction while the other is perpendicular to it); the shape is calculated based on the ratio of these two diameters. (f) The average diameters of CETP, lengths vs. widths. Particle window size is 37 nm in a, b and c. Particle window size is 59 nm in d. All scale bars, 90 nm.

In the present study, CETP was incubated with isolated HDL_2_ at a molar ratio of 1:4 (HDL_2_: CETP), then prepared as described for optimized negative-staining (OpNS) EM method^22^,^23^ (a method that minimizes rouleaux formation), and examined by electron microscopy (EM)^23^,^24^,^25^. A large field EM micrograph and representative particles showed that CETP-HDL_2_ complexes had the appearance of rod shaped CETP penetrating spherical shaped HDL_2_ (Fig. 1a). No CETP was found to bridge two HDL_2_ particles or to adhere to the convex surface of HDL_2_ via its concave surface as hypothesized by crystallography^11^. The HDL_2_ bound to CETP had an average diameter of 12.7 ± 1.6 nm (Fig. 1e), similar to HDL_2_ alone (12.8 ± 1.3 nm) (Supplementary Fig. 1e). The bound CETP particles were 8.7 ± 1.7 nm in length and 3.2 ± 0.5 nm in width (Fig. 1f). Although the width of bound CETP was similar to that of CETP itself (~3.5 nm), the length of the bound CETP was much shorter (~8.7 vs. ~12.5 nm) (Supplementary Fig. 1e), suggesting that ~3–4 nm of the CETP molecule length had been inserted into the HDL_2_ surface (Supplementary Fig. 1e). The EM micrographs and statistical analyses showed that more than 50% of the HDL_2_ bound to CETP, in which, ~30.3% ± 2.7% (mean ± sd) of the HDL_2_ particles bound to one CETP molecule, while 15.7% ± 4.9% (mean ± sd) of the HDL_2_ bound to two CETP molecules and 4.7% ± 4.3% (mean ± sd) of the HDL_2_ bound to more than two CETP molecules. Among these bound HDL_2_, ~40% bound to two or more CETP molecules. In contrast, no CETP molecule bound to more than one HDL_2_ particle simultaneously.

### TEM images of CETP bound to plasma HDL_3_

To investigate how three major HDL surface components: phospholipids, apoA-I and apoA-II affected the CETP binding, the above experiment was repeated with HDL_3_ that contain a different surface protein and lipid ratio than HDL_2_. Earlier studies showed^21^ the HDL_3_ vary in size, but have an average molecular weight of 175 kDa, a density of ~1.125 to ~1.210 g/ml, and contain apoA-I and apoA-II (~55.5% of MW), phospholipids (~22.7% of MW), free cholesterol (~2.8% of MW), cholesterol esters (~14.7% of MW) and triglycerides (~3.4% of MW)^21^. Thus, HDL_3_ has a lower surface percentage of lipids, but a higher surface percentage of proteins than HDL_2_.

In this study, the HDL_3_ sample was incubated with CETP at a molar ratio of 4:1 (CETP:HDL_3_), prepared for OpNS and examined by EM. The EM micrographs showed that the CETP-HDL_3_ complexes were similar to CETP-HDL_2_ complexes, with the rod-shaped CETP adhering to the spherical shaped surface of HDL_3_ (Fig. 1b). As before, no CETP bridged two HDL_3_ particles or adhered to the convex surface of HDL_3_ via its concave surface. The diameter of HDL_3_ that was bound to CETP (Fig. 1e) was ~11.4 ± 1.7 nm, similar to that of HDL_3_ alone (~10.9 ± 1.0 nm) (Supplementary Fig. 1e). The rod shaped CETP in the complexes had a similar width to CETP itself (Supplementary Fig. 1e), but were shorter in length (~8.6 ± 1.2 nm, Fig. 1f). The micrographs and statistical analyses showed more than 40% of the HDL_3_ particles were bound to CETP (compared to ~50% for HDL_2_), in which 32.8% ± 7.0% (mean ± sd) of the HDL_3_ particles bound to one CETP molecule, while 8.2% ± 1.4% (mean ± sd) of the HDL_3_ particles bound to two and more CETP molecules. Among these bound HDL_3_, ~20.2% was also bound to two or more CETP molecules (Fig. 1b). Considering that HDL_3_ and HDL_2_ have the same surface components (apoA-I, apoA-II, and phospholipids), the lower percentages (~41.1% vs. ~50.7%) of CETP bound to HDL that have a lower percentage (22.7% vs. 31.3%) of surface phospholipids suggest that the surface phospholipids/protein ratio may have contributed to some variation in CETP binding.

### TEM images of CETP bound to spherical recombined HDL (rHDL)

To investigate which HDL surface component dominates CETP binding, one of three surface components, apoA-II, was excluded by repeating the above experiment using reconstituted HDL (rHDL)^26^. The rHDL contain apoA-I as the only protein (~43% in MW) and POPC as the only phospholipid (~32.9% in MW). Cholesteryl esters were the only core lipid components (~21.7% in MW)^26^ and a small amount free cholesterol (~2.4%) was distributed between the surface and the core.

In this study, the rHDL sample was incubated with CETP (molar ratio of 4:1 for CETP:rHDL) prepared for OpNS^23^ and examined by EM. The EM micrographs showed ~42.9% of rHDL particles were attached to CETP. No CETP was found to connect two rHDL particles or bind to the rHDL surface via its concave surface. The average diameter of rHDL particles in rHDL-CETP complexes was 10.2 ± 1.2, similar to rHDL (10.3 ± 0.7 nm) alone (Supplementary Fig. 1e). The CETP protrusion length of ~9.2 ± 1.3 nm was shorter than that of CETP alone, but slightly longer than the CETP protrusions on the surfaces of HDL_2_ and HDL_3_ (~9.2 nm vs. ~8.6 nm, Fig. 1f). The micrographs and statistical analyses showed ~34.6% ± 5.3% (mean ± sd) of the rHDL particles bound to one CETP molecule, and ~7.6% ± 3.8% and ~0.7% ± 1.3% (mean ± sd) of the rHDL particles bound to two and more CETP molecules. Approximately 19.4% of the CETP-rHDL complexes were bound to two or more protruding CETP molecules. Notably, most multi-CETP binding complexes (~75%) showed the protruding CETP sharing the same hemisphere of rHDL (Fig. 1c). Moreover, the total number of CETP molecules bound to rHDL can exceed the total number of apoA-I molecules in each rHDL particle (Supplementary Fig. 2), which is consistent to the previous observation^13^. These observations showed that the apoA-II in HDL does not play a significant role in binding to CETP.

### EM images of CETP bound to liposomes

**Figure 2:**
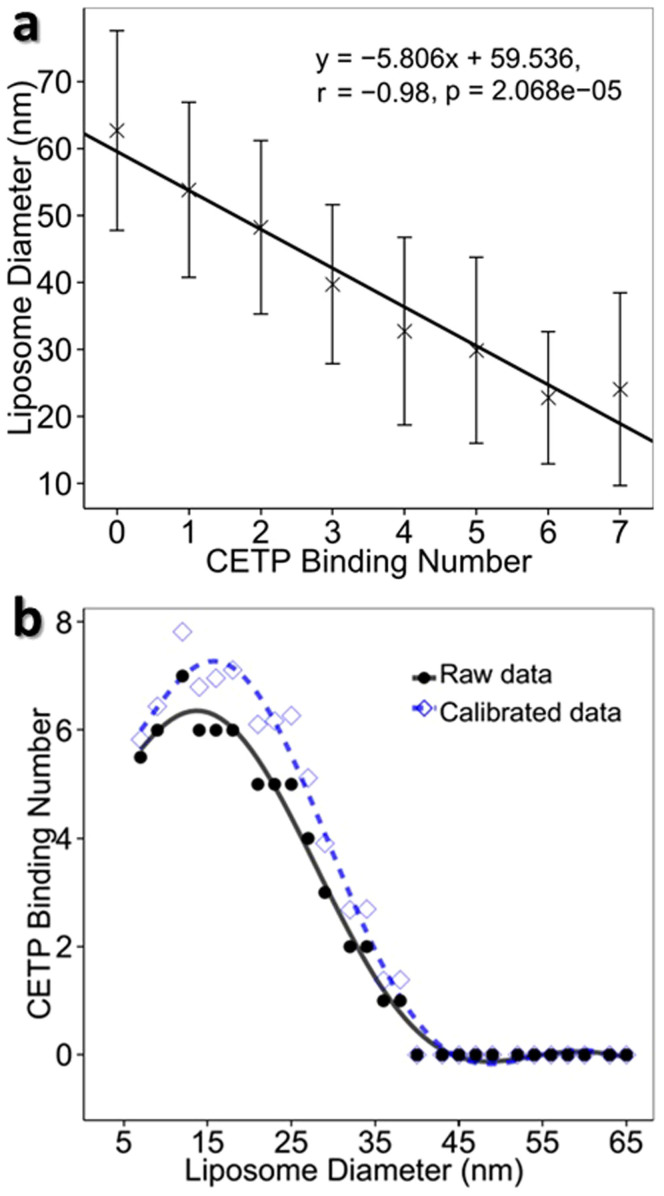
Distributions of the number of bound CETP on liposome against liposome diameter. Panel (a) shows a linear plot of mean of liposome diameters versus the number of its binding CETP molecules. The mean diameter was calculated within each CETP binding number group. The data were approximated by linear regression (R = −0.98, p = 2.06E-05). Panel (b) shows a plot of the mean number of binding CETP molecules versus their binding liposome diameter (black line). As some CETP molecules that are located behind/in-front of the liposomes may not be counted, a geometric model (shown in supplementary Fig. 4) was used to adjust the binding number. The adjusted/calibrated number of bound CETP molecules versus the liposome diameter was also plotted (shown in blue line). The data was fitted with a sixth-degree polynomial function (histograms have a sampling step of 2.17 nm on liposome diameter) using a total of 3,282 liposome/liposome-CETP complexes. Each liposome diameter was calculated as the geometric mean (the square root of the product) of two perpendicular diameters.

To investigate which one of the remaining two HDL surface components, apoA-I or phospholipids, dominates the CETP binding, a lipid vesicle without apoA-I was further used to repeat the above experiments. The lipid vesicle (a POPC-only liposome) was incubated with CETP, and the sample was prepared by OpNS and examined by EM. The EM micrographs and representative liposomes showed the liposomes had a distribution of ~7.7 to ~116.2 nm in diameter (Fig. 1d), which is within the diameter range of all types of lipoproteins (from small HDL to large VLDL). Zoomed in views of a micrograph (Supplementary Fig. 3) and representative particle images (Fig. 1d, top right panel) showed spherical liposomes with surface CETP protrusions with an average length of ~8.5 ± 0.6 nm (Fig. 1f). The liposome particles retained their shape and structure after CETP insertion. Incubation with CETP did not cause particle disruption. Additionally, CETP did not form a bridge between two liposome particles, or bind to a liposome so that its concave surface was adjacent to the convex surface of the liposome. The micrographs and statistical analyses showed ~19.1% ± 3.2% of the liposome particles bound to 1–2 CETP molecules, ~6.0% ± 2.7% of the liposome particles bound to 3–4 CETP molecules, and ~5.1% ± 3.6% and 2.7% ± 1.7% bound to 5–6, or 7 and more CETP molecules respectively. In total, ~32% of the liposomes contained at least one surface CETP protrusion. Among these CETP bound liposomes, ~66% were bound to two or more CETP molecules (Fig. 1d). Notably, smaller liposomes had more CETP molecules bound (Fig. 2). For instance, 5–7 CETP molecules were bound to liposomes ~15 nm in diameter, while liposomes over ~40 nm were rarely bound to CETP (Fig. 2b and Supplementary Fig.4). This relationship was analyzed based on a linear fitting the number of bound CETP against the liposome diameters (Fig. 2a). This result suggests that the smaller liposomes had a higher binding affinity to CETP. Moreover, it confirmed that apoA-I in HDL do not dominate the CETP binding.

### Three dimensional structure of a complex of CETP bound to liposome by individual-particle cryo-electron tomography

**Figure 3:**
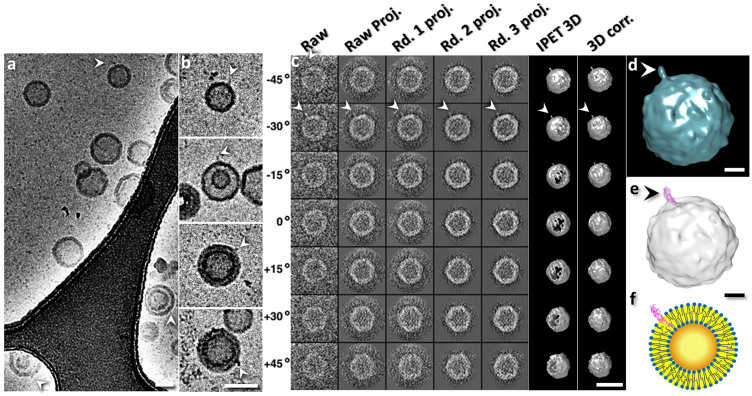
Electron cryo-micrograph (cryo-EM) and 3D reconstruction of a CETP-liposome complex. (a) Cryo-EM images of the sample of POPC liposomes incubated with CETP. (b) Four representative liposome-CETP complexes. (c) *Ab-inito* 3D reconstruction of an individual liposome-CETP complex by individual-particle electron tomography (IPET). The complex was imaged by electron cryo-tomography (cryo-ET, tilting angles ranging from −57° to 60° in steps of 1.5°). Seven representative tilting views (contrast reversed) of a targeted liposome-CETP complex (CTF corrected) were compared to the projections of the intermediate 3D reconstructions (column 2 to 3) and final reconstruction (column 4) at their corresponding angles. The final 3D reconstructions (before and after missing wedge correction) viewing from the corresponding angles were also displayed in last two columns. (d) The final 3D reconstruction of a targeted liposome-CETP complex (resolution of 3.5 nm–7.2 nm based on the Fourier shell correlation, FSC, curve declined to a value of 0.5 and 0.143). (e) Ridge-body docking a CETP crystal structure (PDB ID: 2OBD) into an obvious protrusion of the 3D reconstruction. (f) Schematic of a CETP-liposome complex. The CETP protrusions in all above images were indicated by arrows. Scale Bars: a–c, 50 nm; d, 10 nm.

To confirm that the conformation of liposome-CETP complex was not due to negative-stain artifact, the sample was also flash-frozen in vitreous buffer without any negative-staining. The frozen sample was then examined by cryo-electron microscopy (cryo-EM) at −170°C and under **a** low-dose imaging condition (Fig. 3a). The micrographs and selected particles showed the CETP liposome surfaces adhering with rod-shape CETP (arrows in Fig. 3b), similar to that from the OpNS (Fig. 1d). To reveal the detailed structure in three dimensions (3D), the sample was also imaged by cryo-electron tomography (cryo-ET) through a series of tilting angles (angle range of −57° to 60° in steps of 1.5°). A 3D structure of a representative complex was reconstructed by individual-particle electron tomography (IPET)^27^. IPET was designed for 3D reconstruction of an asymmetric individual particle rather than averaging different particles like in conventional single-particle reconstruction. The naturally varying particle diameter of liposomes made it impossible to achieve any 3D reconstruction by conventional cryo-EM single-particle reconstruction methods.

A total of 79 tilting images of a representative liposome-CETP complex were windowed from CTF-corrected (by TOMOCTF^28^) cryo-ET micrographs (Fig. 3c, left panel). Although the tilt images (but CTF corrected) were noisy, a protrusion of CETP was marginally visible (arrows in Fig. 3c, left panel). To confirm that the protrusion was from a bound CETP instead of noise, three images from consecutively tilted angles (28.5°, 30° and 31.5°) were averaged together to enhance the signal contrast and to reduce the noise level. The averaged image showed the protrusion was more clearly visible (arrow in Supplementary Fig. 5a) than in any individual tilt image, suggesting that the protrusion is indeed from CETP instead of noise. During IPET reconstruction, the tilt images were gradually aligned precisely to their “global center” via an iteration process^27^. The noise in the 3D reconstructions and projections was gradually eliminated as the signal was enhanced (Fig. 3c from second to last column). The protrusion remained visible in each intermediate 3D reconstruction and projection (arrows indicated in Fig. 3c from second to fifth column) as well as the final 3D reconstruction (Fig. 3d). To further confirm whether the protrusion in the final 3D reconstruction was from real signal instead of noise, five central slices (arrows indicated in Supplementary Fig. 5b) of the final 3D reconstruction were directly averaged together. This averaged image and its contour map showed the protrusion remained significant (Supplementary Figs. 5c and d). Fourier shell correlation (FSC) analysis showed the resolution is from ~35 Å to 72 Å based on Supplementary Fig. 6. By rigid-body docking the crystal structure of CETP (PDB entrance: 2OBD^11^) into the protrusion, a near perfect match to the protrusion diameter in width, but ~5 nm shorter in length, suggests a CETP penetrating in the liposome surface (Fig. 3e and f). This experiment confirmed the conformation where CETP penetrates the liposome surface is not due to a negative-staining artifact.

### MD simulation study of the relationship between liposome size and surface hydrophobicity

To understand why CETP interacts with the lipid surface of HDL/liposome without apoA-I or apoA-II (Fig. 1) and why smaller liposomes had a higher binding affinity to CETP (Fig. 2), we hypothesized that a higher surface curvature in a smaller HDL/liposome^29^ will generate a higher degree of surface hydrophobicity (Supplementary Fig. 7b) and increase the affinity of the interaction of the relatively hydrophobic distal end of the CETP N-terminal domain with the liposome surface^13^ (Supplementary Fig. 7a).

**Figure 4:**
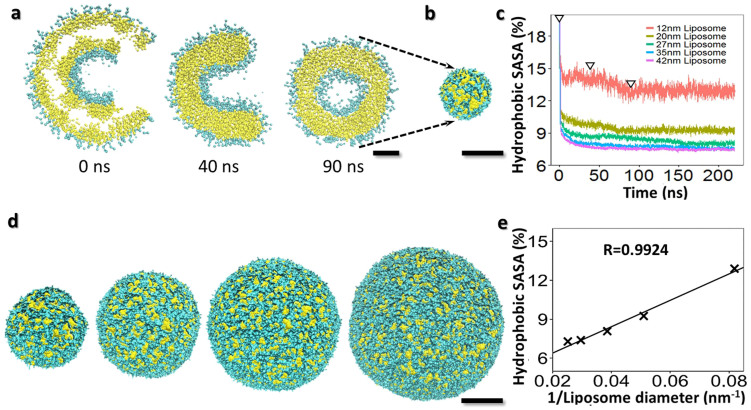
Hydrophobicity analysis of POPC liposome by molecular dynamics (MD) simulation. (a) A cross-section view of initial model and the processes to achieve a 12.2 nm liposome by MD simulations. The initial model (cross-section view, left panel), a phospholipids double-layer sphere with an open pore, was used. The pore allows the outer- and inner-shell phospholipids exchange to maintain the balance between the outer and inner shells during MD simulation. By energy minimization, the pore was gradually closed while the liposome shrank (cross section views, right two panels). (b) After MD simulation for 90 ns, the pore was completely closed, and an energy minimized liposome, 12.2 nm in diameter, was achieved. (c) Plot of the liposome surface hydrophobic area percentage against the MD simulation time. The percentages were computed by taking their hydrophobic surface area divided by the whole liposome surface area. The three triangles shown in the diagram indicated the three intermediate states of the above liposome during energy minimization. (d) Four larger size liposomes with diameters of 19.6 nm, 26.0 nm, 34.4 nm and 42 nm were also achieved by the energy minimization. (e) Plot of the percentages of surface hydrophobic area against the liposome diameters. The data was approximated by linear regression (R = 0.99, p = 7.99E-04). Cyan for the hydrophilic components of POPC molecule and yellow for the hydrophobic lipid tails. Scale Bars: a, 3 nm; b, d and e, 10 nm.

To test this hypothesis, we employed MD simulation to study the liposome surface hydrophobicity against the liposome size by generating a series of liposome vesicles with diameters of ~12 nm, ~20 nm, ~27 nm, ~35 nm and ~42 nm (Fig. 4, and supporting Table 1). After energy minimization (Supplementary Fig. 8), the analyses on the surface hydrophobicity of each lipid vesicle showed that the percentages of surface hydrophobic area (measured by the solvent accessible surface area, SASA^14^) were ~12.9%, ~9.2%, ~8.0%, ~7.4% and ~7.3% respectively (Fig. 4b and d), suggesting that smaller liposomes had a higher hydrophobicity than the larger liposomes (Fig. 4e).

## Discussion

Our experiments showed that, i) the similar morphology in CETP binding between plasma HDL and rHDL implied that the absence of ApoA-II and TG in rHDL did not affect CETP binding to HDL (Fig. 1); ii) the total number of CETP molecules bound to rHDL can exceed the total number of apoA-I molecules in each rHDL particle; iii) non-apoA-I containing liposomes showed similar CETP protrusions to apoA-I containing HDL (Fig. 1d); iv) that there is a significant correlation between liposome size and number of bound CETP molecules; and v) a significant correlation between liposome size and the percentage of surface hydrophobic area. Those results suggest that apoA-I and apoA-II may not be involved in CETP binding; however, the surface phospholipids, and surface curvature likely dominate the interaction of CETP with the surface of HDL.

A larger surface lipid area provides a higher opportunity for CETP binding. We noticed that HDL_2_, which has has a larger diameter than HDL_3_ (~13 vs. ~11), also has a higher percentage of bound CETP particles than HDL_3_ (~50% vs. ~42%). This may due to the HDL_2_ surface lipids occupying a larger area than the surface lipids of HDL_3_ (~31.3% vs. ~22.7%). This is consistent with liposomes which are larger than HDL2 or HDL3 having ~6 bound CETP molecules (Fig. 1d and 2b), compared to the average of 1–2 CETP molecules that are bound to HDL_2_ and HDL_3_ (Fig. 1a and b). This suggests that the binding affinity of CETP is regulated by surface lipid area, as well as surface curvature.

Our results favor the tunnel mechanism rather than the shuttle mechanism for the transfer of CE from HDL to LDL via CETP. Both of these mechanisms were hypothesized to be involved in the CETP-mediated transfer of CE between HDL and LDL two decades ago^30^,^31^. In the shuttle mechanism, CETP interacts with HDL and acquires CEs, after which the “CE-enriched” CETP is released from the HDL for subsequent deposition into LDL. The “CE-poor” CETP is then released from the LDL surface for subsequent interaction with HDL in another cycle of CE transfer^32^. In the tunnel mechanism, CETP binds to HDL and LDL simultaneously to form a ternary complex. CETP then undergoes a conformational change to form a hydrophobic tunnel whereby CEs transfer from HDL to LDL.

Our results showed that CETP bound to only one HDL particle at a time, and did not form a bridge between two HDL_2_ HDL_3_ or rHDL particles. This suggests that the binding of CETP to HDL is directional. When the fact that i) we were unable to demonstrate CETP bridging two HDL particles (Fig. 1), ii)small HDL/liposome particles with a higher surface curvature and hydrophobicity are more likely to bind to more CETP molecules (Fig. 1 and 2), iii) the N-terminal β-barrel domain of CETP binds to HDL while the C-terminal β-barrel domain binds to LDL^13^, and iv) the distal end of the N-terminal β-barrel domain of CETP is relatively hydrophobic^11^, are considered in light of the current results which show that CETP binds to only one liposome particle at a time, and does not bridge two liposomes (Fig. 1, 2 and 3), it follows that the distal end of the CETP N-terminal β-barrel domain is most likely inserted into the liposome particle surface via a protein-lipid interaction. This interaction is different for LDL particles, where the distal end of the C-terminal β-barrel domain of CETP inserts to the LDL surface^13^, possibly via a protein-protein interaction. The conclusion that there is a reduced interaction of the distal end of the N-terminal domain of CETP with LDL surface lipids may be due to the fact that LDL particles have a flattened, ellipsoidal shape with planar opposing faces that, as shown in our earlier cryo-EM single-particle reconstruction^33^, are largely covered by apoB-100 (~500 kDa). The reduced lipid surface area combined with the reduced surface curvature of the particles is likely to have contributed to the low binding affinity of the hydrophobic distal end of the CETP N-terminal β-barrel domain to LDL.

CETP directionally bound to HDL is consistent with many earlier reports, including i) a CETP-mediated net transfer of CE into VLDL^34^, ii) CETP bridging HDL and LDL instead of bridging two HDL or LDL particles^13^, iii) the distal portion of the N-terminal β-barrel domain of CETP being more flexible in solution than is indicated by its crystal structure^14^; and iv) MD simulations showing that the penetration of the N-terminal β-barrel domain of CETP into the HDL surface generates an opening that allows CE to access the CETP tunnel^15^. However, our results are inconsistent with those of other investigators who have shown that CETP binds to the edge of discoidal HDL^35^ via an interaction with apoA-I^36^, and atomistic and coarse-grained simulations of CETP–HDL interaction which have shown that the N-terminus helix-X domain of CETP penetrates deeply into the HDL particle core^37^. Nonetheless, our results favor a tunnel, not a shuttle mechanism whereby CETP no longer binds to HDL when it has acquired an appropriate number of CEs from HDL.

While we have illuminated some of the mechanism behind CETP, several questions remain: i) how the CETP hydrophobic N-terminal β-barrel domain of CETP penetrates the HDL surface and with such high specificity (e.g. not using the C-term β-barrel domain? Normally, as hydrophobic interactions are not specific, it is unclear how much this affinity relate to the surface curvature interactions in concert with the N-term distal end to open up); ii) how CE molecules can be transferred through a ~10 nm channel; iii) how TGs can be transferred back to HDL from LDL; iv) how CE and TG exchange between LDL and VLDL; v) how CETP homo-exchanged the radiolabeled lipid transfer among HDL particles. Our results highlight the CETP N-terminal β-barrel domain hydrophobic distal end as a potential drug target, which may lead to a next-generation drug to treat CVDs.

## Methods

### Protein and liposome isolation

Recombinant human CETP (~53 kDa with no post-translational modifications) was expressed and purified from the Chinese hamster ovary cell line DG441, as previously described^11^. The CETP concentration was determined by absorbance at 280 nm. Native plasma HDL_2_ and HDL_3_ in phosphate buffered saline (PBS) were isolated from fresh, pooled samples of human plasma by ultracentrifugation as reported^38^. Pooled samples from multiple donors were used to make sure that the sample was representative and that the results could not be attributed to something that specifically related to a single individual. Spherical, reconstituted HDL (rHDL) in PBS were prepared^39^,^40^. Liposome vesicle samples were produced by Encapsula NanoSciences (Brentwood, TN). The sample contained 1 mg/ml 1-Palmitoyl-2-oleoylphosphatidylcholine (POPC, from Avanti Polar lipids) with a peak vesicle size of ~50 nm in a buffer containing 20 mM Tris-Cl, 154 mM NaCl, pH 7.4.

### Specimen preparation for negative-staining EM

Specimens were prepared for EM using the optimized negative-staining (OpNS) protocol^23^,^25^, which minimizes the rouleaux artifact that is observed with lipoproteins^23^,^24^,^25^. In brief, CETP (0.28 mg/ml) was incubated with HDL_2_, HDL_3_, rHDL and liposomes, respectively at 37°C for 15 minutes at a molar ratio of ~4:1 (CETP: lipid macromolecular particle). CETP-lipid macromolecular complexes were diluted to 1.5 μg/ml with Dulbecco's phosphate buffered saline (DPBS). A 4 μl aliquot was placed on a thin-carbon-coated copper grid (300 mesh TEM grid, Cu-300CN, Pacific Grid-Tech, San Francisco, CA) that had been glow-discharged. After one minute, excess solution was blotted with filter paper, followed by washing and negative staining with 1% (w/v) uranyl formate (UF)^23^,^25^. After air drying, the grids were further dried for an hour at 40°C.

### Electron microscopy data acquisition and image pre-processing

The OpNS micrographs were acquired under defocus between ~0.6 um to ~2.2 um on a Gatan UltraScan 4 K × 4 K CCD equipped on a Zeiss Libra 120 Plus transmission electron microscope (Carl Zeiss NTS GmbH, Oberkochen, Germany). The TEM was operated under a high-tension of 120 kV, energy filtering of 20 eV and magnification range of 31.5 K to 80 K, in which each pixel of the micrographs corresponded to 3.68 to 1.48 Å respectively. A total of 15–72 micrographs were imaged from each sample. The contrast transfer function (CTF) of each micrograph was determined and then corrected by the phase-flip option using *ctfit* (EMAN software)^41^. ~300–~3000 particles from each sample were selected and windowed by the *boxer* software in the EMAN software package^41^ and submitted for Gaussian low-pass filtering before size measurement.

### Cryo-electron microscopy (cryo-EM) and cryo-electron tomography (cryo-ET) data acquisition

Cryo-EM specimens were prepared on lacey carbon film coated copper grid (Cu-200LC, Pacific Grid-Tech, San Francisco, CA). Cryo-EM data of liposome binding to CETP specimens were acquired under a less than ~2.3 μm defocus with a high-sensitivity 4 K × 4 K pixel Gatan Ultrascan CCD camera at 50 K magnification by the same Zeiss Libra 120 TEM (each pixel of the micrograph corresponded to 2.4 Å in the specimens). Total dose for un-tilt 2D micrographs is about 10 to 30 e^−^/Å^2^. For cryo-ET data acquisition, the specimens mounted on a Gatan 915 high-tilt cryo-EM holder were tilted at angles ranging from −57° to 60° in steps of 1.5°. The total dose of electron illumination was up to ~120 e^−^/Å^2^ or slightly higher. The tilt series of tomographic data was controlled and imaged by manual operation and by Gatan tomography software (Zeiss Libra 120 TEM) that was preinstalled in the microscope.

### Correction of contrast transfer function (CTF) for cryo-ET data

Tilting series of micrographs were initially aligned together with the IMOD software package^42^. The defocus near the tilt-axis area of each tilt micrograph was examined by fitting CTF parameters with its power spectrum by *ctffind3* in the FREALIGN software package^43^ and then examined by *ctfit* (EMAN software package)^41^. The CTF was then corrected by TOMOCTF^28^. The tilt series of each CETP-liposome image in windows of 200 × 200 pixels was tracked and selected by IPET software.

### Individual-particle electron tomography (IPET) 3D reconstruction

*Ab-initio* 3D reconstruction of an individual liposome-CETP complex was conducted by the IPET method^27^. In IPET, a small image containing only a targeted liposome-CETP complex was windowed from each tilted whole-micrograph (CTF corrected). An *ab-initio* initial model was generated by directly back-projecting these small-images into a 3D map. The map was then refined via three rounds of refinement loops (including more than a hundred iterations) by the focused electron tomography reconstruction (FETR) algorithm^27^. In FETR, an automatically-generated dynamic Gaussian low-pass filter and an automatically generated soft-mask were applied to both the references and tilted images to achieve the final 3D reconstruction. Since the specimen holder has a limitation to tilt to ±90° angle, a wedge shaped data was missing in final 3D reconstruction, which resulted in a certain level of artifact, especially along the Z direction. The missing wedge data was estimated via our newly developed interactive algorithm (related manuscript in preparation). As an implementation of this algorithm, we computed the missing wedge data and contributed to the final reconstruction. The crystal structure (PDB entry 2OBD^11^) was fitted into the final IPET 3D reconstruction by using a rigid-body fitting option in UCSF Chimera^44^.

### Fourier shell correlation (FSC) analysis

To analyze tomographic 3D reconstruction resolution, the center-refined raw ET images were split into two groups based on having an odd- or even-numbered index in the order of tilt angles^27^. Each group was used to generate a 3D reconstruction image; the two 3D reconstructions from both groups were then used to compute the FSC curve over their corresponding spatial frequency shells in Fourier space (using the “RF 3” command in SPIDER^45^) (Supplementary Fig. 5). The frequency at which the FSC curve declined to a value of 0.5 and 0.143 (golden standard^46^) was used to represent the resolution of the final reconstruction.

### Statistical analyses of CETP binding to HDL particles and liposomes

Particle size was determined by measuring the diameter in two orthogonal directions, as described before^25^. In brief, the geometric mean of the perpendicular diameters was used to represent the particle diameter. The aspect ratio of the long and perpendicular diameters was used to represent particle shape. Histograms of the particle diameters were generated with 2.17 nm sampling steps. Each histogram was fitted with a 9^th^ degree polynomial function in R for data analysis. Since the CETP bound to the liposome may have been blocked from certain viewing directions, we computed the probability based a geometric model (Supplementary Fig. 3). The probability (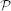) is a function of the liposome diameter (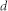) and CETP protrusion length (*l*), in equation of 
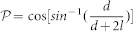
. The final histogram was adjusted by taking the measured histogram divided by this variable probability.

### Liposome structures by molecular dynamics (MD) simulation and hydrophobicity analysis

Based on the size range of liposomes in our experiment, five initial liposome models with diameters of 12 nm, 20 nm, 27 nm, 35 nm and 42 nm were simulated. The initial models to simulate the liposomes were generated as below, the lipid bilayer vesicles (~65 Å^2^/lipids^47^ and the average thickness ~36.8 Å^48^) with an open pore (with 13% of surface area)^49^ were surrounded by a solution containing water molecules and NaCl at 0.1 M. These pores on the vesicles would allow the lipid flipping and transferring between inner and outer membranes during energy minimization and MD simulations^50^,^51^.

As the liposomes contained a large number of molecules, we used the Residue-based Coarse Graining (RBCG) MD method to simplify our initial models in order to enable a long time-scale simulation^52^. In RBCG, about every 10 atoms were grouped together and assigned as a bead model based on their chemical functionality. One POPC molecule was assigned 13 beads (one bead for choline, one for phosphate, two beads for glycerol and nine for two fatty acid chains) based on Marrink's CG lipid model^53^. The interaction potentials between beads and the bead masses were used as previously reported^54^.

Energy minimization was conducted by Nanoscale Molecular Dynamics (NAMD)^55^. The criteria to determine whether the liposomes were stabilized were as follows: i) the surface pores were completely closed; ii) liposomes should have a spherical shape determined by Radial Distribution Function (RDF), which is computed with respect to the liposome's center of mass (supplementary Fig. 8); and iii) stabilized surface hydrophobicity. The area of the hydrophobic surface on each stabilized liposome was measured by the Solvent Accessible Surface Area (SASA) function in Visual Molecular Dynamics (VMD)^56^. The percentages of surface hydrophobic area on each liposome surface were computed by taking their hydrophobic surface area and dividing by the whole liposome surface area. The average of the last 300 frames (12 ns) was used to calculate the hydrophobicity.

## Author Contributions

This project was initiated and designed by R.C., L.Z., K.A.R., X.Q., D.G.J. and G.R.; and refined by M.Z. and M.A.C.; X.Q., D.G.J., M.P. and K.A.R. isolated the CETP and HDL samples; M.Z., L.Z. and R.C. conducted the experiments and acquired the OpNS data; H.T. and L.Z. acquired the cryo-EM data; M.Z. processed the NS data, computed the statistics and performed the molecular dynamics simulation;L.Z., M.Z. and F.W. processed the cryo-ET data; A.R. analyzed the projections and averages of 3D reconstruction; M.Z., L.Z., R.C., M.A.C., M.J.R. and G.R. interpreted and manipulated the data; M.Z., R.C., M.A.C. and G.R. drafted the initial manuscript, which was revised by L.Z., K.A.R., A.R., M.P., M.J.R., X.Q. and D.G.J.

## Supplementary Material

Supplementary Information Supporting info

## References

[b1] Drayna D. *et al.* Cloning and sequencing of human cholesteryl ester transfer protein cDNA. Nature 327, 632–4 (1987). 3600759 10.1038/327632a0

[b2] Inazu A. *et al.* Increased high-density lipoprotein levels caused by a common cholesteryl-ester transfer protein gene mutation. N Engl J Med 323, 1234–8 (1990). 2215607 10.1056/NEJM199011013231803

[b3] Brown M. L. *et al.* Molecular basis of lipid transfer protein deficiency in a family with increased high-density lipoproteins. Nature 342, 448–51 (1989). 2586614 10.1038/342448a0

[b4] Zhong S. *et al.* Increased coronary heart disease in Japanese-American men with mutation in the cholesteryl ester transfer protein gene despite increased HDL levels. J Clin Invest 97, 2917–23 (1996). 8675707 10.1172/JCI118751 PMC507389

[b5] Barter P. J. *et al.* Effects of torcetrapib in patients at high risk for coronary events. N Engl J Med 357, 2109–22 (2007). 17984165 10.1056/NEJMoa0706628

[b6] Funder J. W. The off-target effects of torcetrapib. Endocrinology 150, 2024–6 (2009). 19383878 10.1210/en.2009-0136

[b7] Stein E. A. *et al.* Safety and tolerability of dalcetrapib. Am J Cardiol 104, 82–91 (2009). 19576325 10.1016/j.amjcard.2009.02.061

[b8] Derks M., Anzures-Cabrera J., Turnbull L. & Phelan M. Safety, tolerability and pharmacokinetics of dalcetrapib following single and multiple ascending doses in healthy subjects: a randomized, double-blind, placebo-controlled, phase I study. Clin Drug Investig 31, 325–35 (2011). 10.1007/BF03256931 21366361

[b9] Gotto A. M. Jr *et al.* Evaluation of lipids, drug concentration, and safety parameters following cessation of treatment with the cholesteryl ester transfer protein inhibitor anacetrapib in patients with or at high risk for coronary heart disease. Am J Cardiol 113, 76–83 (2014). 24188894 10.1016/j.amjcard.2013.08.041

[b10] Friedrich S. *et al.* The pharmacokinetics and pharmacokinetic/pharmacodynamic relationships of evacetrapib administered as monotherapy or in combination with statins. CPT Pharmacometrics Syst Pharmacol 3, e94 (2014). 24452615 10.1038/psp.2013.70 PMC3910017

[b11] Qiu X. *et al.* Crystal structure of cholesteryl ester transfer protein reveals a long tunnel and four bound lipid molecules. Nat Struct Mol Biol 14, 106–13 (2007). 17237796 10.1038/nsmb1197

[b12] Liu S. *et al.* Crystal structures of cholesteryl ester transfer protein in complex with inhibitors. J Biol Chem 287, 37321–9 (2012). 22961980 10.1074/jbc.M112.380063 PMC3481329

[b13] Zhang L. *et al.* Structural basis of transfer between lipoproteins by cholesteryl ester transfer protein. Nat Chem Biol 8, 342–9 (2012). 22344176 10.1038/nchembio.796 PMC3792710

[b14] Lei D. *et al.* Structural features of cholesteryl ester transfer protein: a molecular dynamics simulation study. Proteins 81, 415–25 (2013). 23042613 10.1002/prot.24200 PMC3557553

[b15] Cilpa-Karhu G., Jauhiainen M. & Riekkola M. L. Atomistic molecular dynamics simulation reveals the mechanism by which CETP penetrates into HDL enabling lipid transfer from HDL to CETP. J Lipid Res (2014). 10.1194/jlr.M054288 PMC4274075 25424006

[b16] van Antwerpen R., La Belle M., Navratilova E. & Krauss R. M. Structural heterogeneity of apoB-containing serum lipoproteins visualized using cryo-electron microscopy. J Lipid Res 40, 1827–36 (1999). 10508202

[b17] van Antwerpen R. *et al.* Cryo-electron microscopy of low density lipoprotein and reconstituted discoidal high density lipoprotein: imaging of the apolipoprotein moiety. J Lipid Res 38, 659–69 (1997). 9144081

[b18] Pattnaik N. M. & Zilversmit D. B. Interaction of cholesteryl ester exchange protein with human plasma lipoproteins and phospholipid vesicles. J Biol Chem 254, 2782–6 (1979). 34609

[b19] Jeyarajah E. J., Cromwell W. C. & Otvos J. D. Lipoprotein particle analysis by nuclear magnetic resonance spectroscopy. Clin Lab Med 26, 847–70 (2006). 17110242 10.1016/j.cll.2006.07.006

[b20] Otvos J. D., Jeyarajah E. J. & Cromwell W. C. Measurement issues related to lipoprotein heterogeneity. Am J Cardiol 90, 22i–29i (2002). 10.1016/s0002-9149(02)02632-2 12419478

[b21] Lund-Katz S., Liu L., Thuahnai S. T. & Phillips M. C. High density lipoprotein structure. Front Biosci 8, d1044–54 (2003). 12700101 10.2741/1077

[b22] Orlova E. V. *et al.* Three-dimensional structure of low density lipoproteins by electron cryomicroscopy. Proc Natl Acad Sci U S A 96, 8420–5 (1999). 10411890 10.1073/pnas.96.15.8420 PMC17531

[b23] Zhang L. *et al.* An optimized negative-staining protocol of electron microscopy for apoE4 POPC lipoprotein. J Lipid Res 51, 1228–36 (2010). 19965615 10.1194/jlr.D002493 PMC2853450

[b24] Zhang L., Tong H., Garewal M. & Ren G. Optimized negative-staining electron microscopy for lipoprotein studies. Biochim Biophys Acta 1830, 2150–9 (2013). 23032862 10.1016/j.bbagen.2012.09.016 PMC3508368

[b25] Zhang L. *et al.* Morphology and structure of lipoproteins revealed by an optimized negative-staining protocol of electron microscopy. J Lipid Res 52, 175–84 (2011). 20978167 10.1194/jlr.D010959 PMC2999936

[b26] Zhang B. *et al.* Effects of reconstituted HDL on charge-based LDL subfractions as characterized by capillary isotachophoresis. J Lipid Res 48, 1175–89 (2007). 17327623 10.1194/jlr.M600227-JLR200

[b27] Zhang L. & Ren G. IPET and FETR: experimental approach for studying molecular structure dynamics by cryo-electron tomography of a single-molecule structure. PLoS One 7, e30249 (2012). 22291925 10.1371/journal.pone.0030249 PMC3265479

[b28] Fernandez J. J., Li S. & Crowther R. A. CTF determination and correction in electron cryotomography. Ultramicroscopy 106, 587–96 (2006). 16616422 10.1016/j.ultramic.2006.02.004

[b29] Jensen M. B. *et al.* Membrane curvature sensing by amphipathic helices: a single liposome study using alpha-synuclein and annexin B12. J Biol Chem 286, 42603–14 (2011). 21953452 10.1074/jbc.M111.271130 PMC3234936

[b30] Barter P. J. & Jones M. E. Kinetic studies of the transfer of esterified cholesterol between human plasma low and high density lipoproteins. J Lipid Res 21, 238–49 (1980). 7373163

[b31] Ihm J., Quinn D. M., Busch S. J., Chataing B. & Harmony J. A. Kinetics of plasma protein-catalyzed exchange of phosphatidylcholine and cholesteryl ester between plasma lipoproteins. J Lipid Res 23, 1328–41 (1982). 7161562

[b32] Tall A. R. Plasma cholesteryl ester transfer protein. J Lipid Res 34, 1255–74 (1993). 8409761

[b33] Ren G. *et al.* Model of human low-density lipoprotein and bound receptor based on cryoEM. Proc Natl Acad Sci U S A 107, 1059–64 (2010). 20080547 10.1073/pnas.0908004107 PMC2798884

[b34] Morton R. E. & Zilversmit D. B. Inter-relationship of lipids transferred by the lipid-transfer protein isolated from human lipoprotein-deficient plasma. J Biol Chem 258, 11751–7 (1983). 6619141

[b35] Bruce C. *et al.* Molecular determinants of plasma cholesteryl ester transfer protein binding to high density lipoproteins. J Biol Chem 270, 11532–42 (1995). 7744792 10.1074/jbc.270.19.11532

[b36] Dergunov A. D., Shabrova E. V. & Dobretsov G. E. Cholesteryl ester diffusion, location and self-association constraints determine CETP activity with discoidal HDL: Excimer probe study. Arch Biochem Biophys 564, 211–8 (2014). 25449063 10.1016/j.abb.2014.09.019

[b37] Koivuniemi A., Vuorela T., Kovanen P. T., Vattulainen I. & Hyvonen M. T. Lipid exchange mechanism of the cholesteryl ester transfer protein clarified by atomistic and coarse-grained simulations. PLoS Comput Biol 8, e1002299 (2012). 22253581 10.1371/journal.pcbi.1002299 PMC3257282

[b38] Han S. *et al.* Comparison of lipoprotein separation and lipid analysis methodologies for human and cynomolgus monkey plasma samples. J Cardiovasc Transl Res 5, 75–83 (2012). 22194019 10.1007/s12265-011-9340-9

[b39] Rye K. A., Hime N. J. & Barter P. J. Evidence that cholesteryl ester transfer protein-mediated reductions in reconstituted high density lipoprotein size involve particle fusion. J Biol Chem 272, 3953–60 (1997). 9020099 10.1074/jbc.272.7.3953

[b40] Rye K. A. & Barter P. J. The influence of apolipoproteins on the structure and function of spheroidal, reconstituted high density lipoproteins. J Biol Chem 269, 10298–303 (1994). 8144610

[b41] Ludtke S. J., Baldwin P. R. & Chiu W. EMAN: semiautomated software for high-resolution single-particle reconstructions. J Struct Biol 128, 82–97 (1999). 10600563 10.1006/jsbi.1999.4174

[b42] Kremer J. R., Mastronarde D. N. & McIntosh J. R. Computer visualization of three-dimensional image data using IMOD. J Struct Biol 116, 71–6 (1996). 8742726 10.1006/jsbi.1996.0013

[b43] Mindell J. A. & Grigorieff N. Accurate determination of local defocus and specimen tilt in electron microscopy. J Struct Biol 142, 334–47 (2003). 12781660 10.1016/s1047-8477(03)00069-8

[b44] Pettersen E. F. *et al.* UCSF Chimera--a visualization system for exploratory research and analysis. J Comput Chem 25, 1605–12 (2004). 15264254 10.1002/jcc.20084

[b45] Frank J. *et al.* SPIDER and WEB: processing and visualization of images in 3D electron microscopy and related fields. J Struct Biol 116, 190–9 (1996). 8742743 10.1006/jsbi.1996.0030

[b46] Scheres S. H. & Chen S. Prevention of overfitting in cryo-EM structure determination. Nat Methods 9, 853–4 (2012). 22842542 10.1038/nmeth.2115 PMC4912033

[b47] Kucerka N., Nieh M. P. & Katsaras J. Fluid phase lipid areas and bilayer thicknesses of commonly used phosphatidylcholines as a function of temperature. Biochim Biophys Acta 1808, 2761–71 (2011). 21819968 10.1016/j.bbamem.2011.07.022

[b48] Tahara Y. & Fujiyoshi Y. A new method to measure bilayer thickness: cryo-electron microscopy of frozen hydrated liposomes and image simulation. Micron 25, 141–9 (1994). 8055245 10.1016/0968-4328(94)90039-6

[b49] Risselada H. J. & Marrink S. J. Curvature effects on lipid packing and dynamics in liposomes revealed by coarse grained molecular dynamics simulations. Phys Chem Chem Phys 11, 2056–67 (2009). 19280016 10.1039/b818782g

[b50] de Vries A. H., Mark A. E. & Marrink S. J. Molecular dynamics simulation of the spontaneous formation of a small DPPC vesicle in water in atomistic detail. J Am Chem Soc 126, 4488–9 (2004). 15070345 10.1021/ja0398417

[b51] Marrink S. J. & Mark A. E. Molecular dynamics simulation of the formation, structure, and dynamics of small phospholipid vesicles. J Am Chem Soc 125, 15233–42 (2003). 14653758 10.1021/ja0352092

[b52] Marrink S. J., de Vries A. H. & Mark A. E. Coarse grained model for semiquantitative lipid simulations. Journal of Physical Chemistry B 108, 750–760 (2004).

[b53] Marrink S. J., Risselada J. & Mark A. E. Simulation of gel phase formation and melting in lipid bilayers using a coarse grained model. Chem Phys Lipids 135, 223–44 (2005). 15921980 10.1016/j.chemphyslip.2005.03.001

[b54] Shih A. Y., Freddolino P. L., Arkhipov A. & Schulten K. Assembly of lipoprotein particles revealed by coarse-grained molecular dynamics simulations. J Struct Biol 157, 579–92 (2007). 17070069 10.1016/j.jsb.2006.08.006

[b55] Phillips J. C. *et al.* Scalable molecular dynamics with NAMD. J Comput Chem 26, 1781–802 (2005). 16222654 10.1002/jcc.20289 PMC2486339

[b56] Humphrey W., Dalke A. & Schulten K. VMD: visual molecular dynamics. J Mol Graph 14, 33–8, 27–8 (1996). 8744570 10.1016/0263-7855(96)00018-5

