# Supplementary material for: HDL surface lipids mediate CETP binding as revealed by electron microscopy and molecular dynamics simulation
Source: Sci Rep. 2015 Mar 4;5:8741. doi: 10.1038/srep08741 (PMC4348656; doi:10.1038/srep08741)
Supplement: Supplementary Information — Supporting info [file srep08741-s1.pdf]

HDL surface lipids mediate CETP binding as revealed by electron microscopy and molecular dynamics simulation

Meng Zhang[1], River Charles[1], Huimin Tong[1], Lei Zhang[1], Mili Patel[2], Francis Wang[1], Matthew J. Rames[1], Amy Ren[1], Kerry-Anne Rye[2], Xiayang Qiu[3], Douglas G. Johns[4], M Arthur Charles[5], Gang Ren[1,*]

[1] The Molecular Foundry, Lawrence Berkeley National Laboratory, Berkeley, CA 94720,
[2] Centre for Vascular Research, University of New South Wales, Kensington, Sydney, NSW 2052, Australia
[3] Pfizer Inc., Groton, Connecticut 06340, USA
[4] Merck Research Laboratories, Rahway, New Jersey 07065, USA
[5] School of Medicine, University of California, San Francisco, California 94115, USA

* Correspondence should be addressed to: G. R. (gren@lbl.gov)

Supplementary Figures

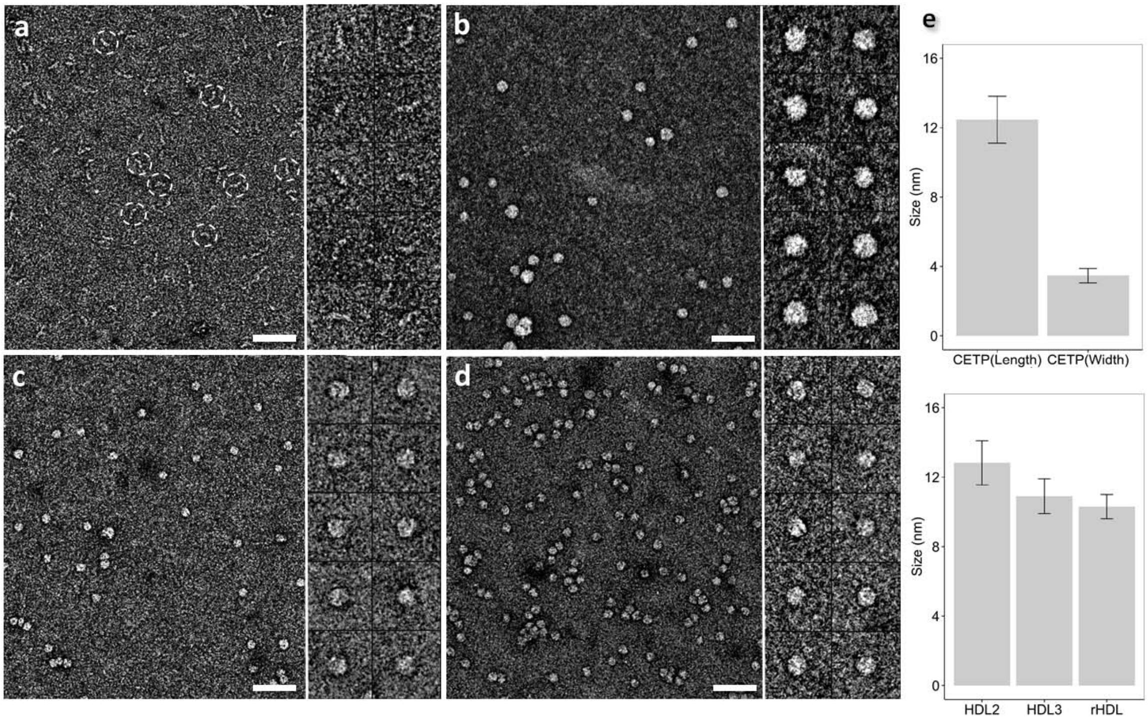

Supplementary Fig. 1: Morphology of CETP, plasma HDL_3, HDL_2 and recombinant spherical HDL (rHDL) by optimized negative-staining (OpNS). (**a**) Survey view (left panel), representative views of raw particles (right panel) of CETP alone (indicated by white circles; (**b**) plasma HDL_2 alone; (**c**) plasma HDL_3 alone; and (**d**) rHDL alone. (**e**) Statistical analyses of the particle size (geometric mean). Bar is 50 nm; Particle window size is 37 nm.

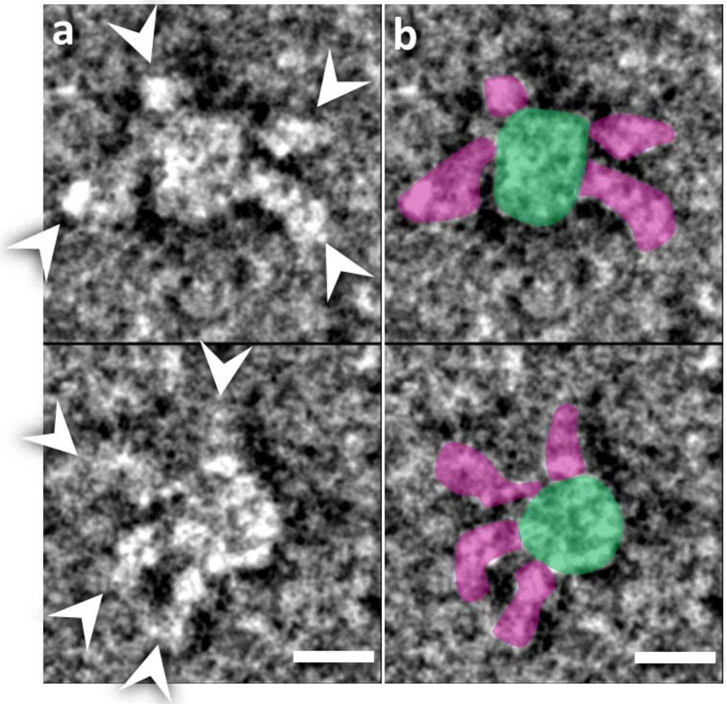

Supplementary Fig. 2: More than four CETPs could bind to one rHDL particle. (a) Two representative particles images and **(b)** their corresponding carton images showed one HDL particle (colored in green) bound to more than three CETP molecules (colored in purple); the number could be more than the copies of apoA-I containing in rHDL particle (3 apoA-I). Bar is 5 nm.

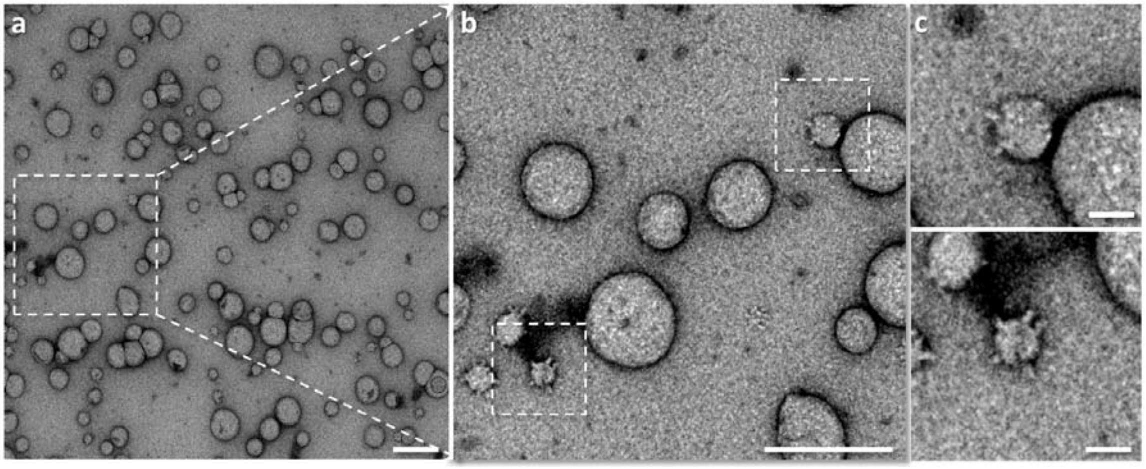

Supplementary Fig. 3: Images of liposome-CETP complexes by OpNS. (**a**) Survey view of the sample of POPC liposome incubated with CETP, (**b** and **c**) Zoomed-in views of the liposome particles bound with multiple CETPs. Scale Bars: a and b, 110 nm; c, 20 nm.

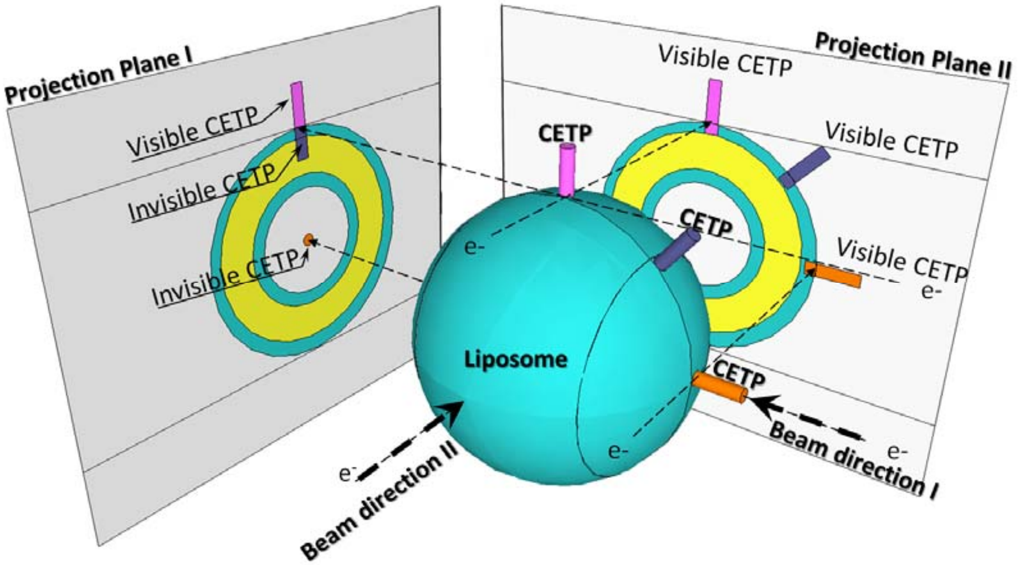

Supplementary Fig. 4: Schematic of a geometric model for computing the probability of CETP visualization on a liposome. The CETP bound to liposomes were only visible from certain viewing angles on the liposome surface. In other words, a CETP bound to a liposome may not be visible by EM due to being behind/in-front of the liposome surface. A geometric model was generated to calculate the probability of visualization, which depended on the liposome diameter (d) and CETP protrusion length (l). This probability given by $\mathcal{P} = \cos\left[sin^{-1}\left(\frac{d}{d+2l}\right)\right]$ was used to adjust the measured histogram.

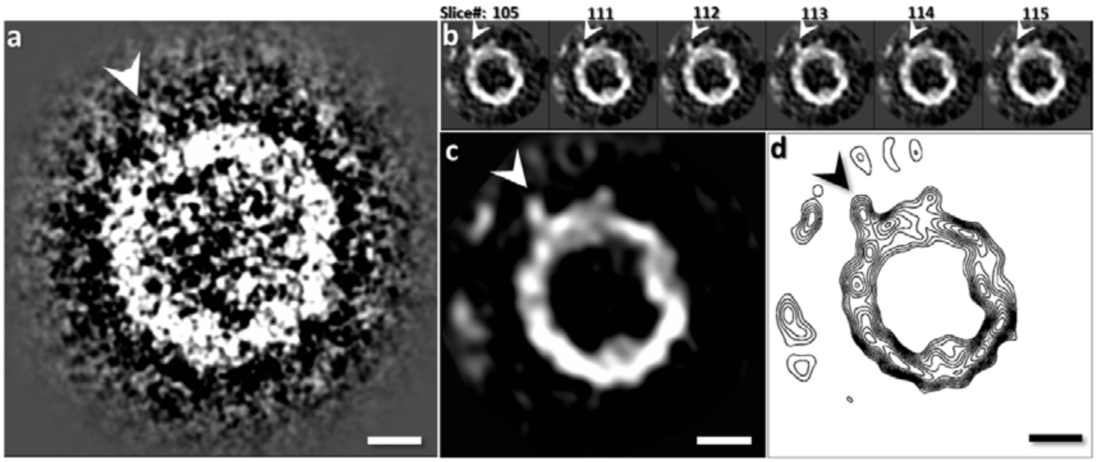

Supplementary Fig. 5: Averaging analysis of the images of CETP protrusion on a liposome surface.(**a**) Average of three sequential tilting images (28.5°, 30.0° and 31.5°), (**b**) Five central slices of the final 3D reconstruction (slice # 110 to #114, 2.4Å/slice), (**c**) the average of the five slices, and (**d**) the average displayed in a contour map (density range from 0.01 to 0.096 in steps of 0.008). The CETP protrusions are indicated by white arrows. Scale Bars: a, 8 nm; c and d, 11 nm.

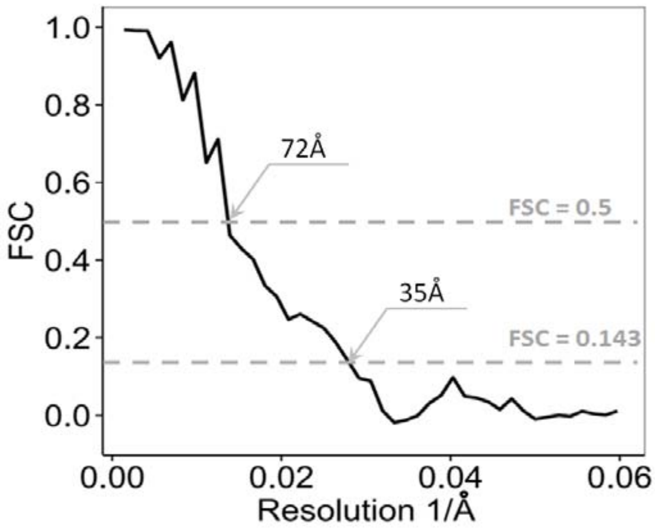

Supplementary Fig. 6: Fourier shell correlation (FSC) analysis of the 3D reconstruction resolution of a liposome-CETP complex. The center-aligned ET images were split into two groups based on having an odd- or even-numbered index in the order of tilt angles. Two 3D reconstructions were generated from each group of tilt images. FSC curve was computed between these two 3D reconstructions. The frequency at which the FSC curve declined to a value of 0.5 and 0.143 was used to represent the resolution of the final reconstruction.

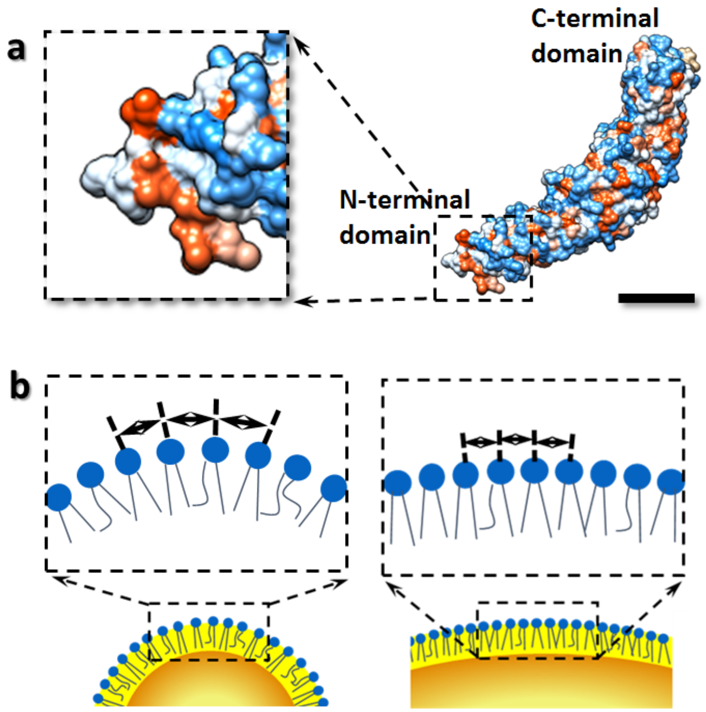

Supplementary Fig. 7: Analyses of hydrophobic surfaces of CETP and liposome. (**a**) The CETP crystal structure is shown according to its van der Waals surface and is colored by its hydrophobicity (ranging from royal blue for the most hydrophilic to orange-red for the most hydrophobic). The N-terminal β-barrel domain showed the distal end has a relatively high level of hydrophobicity. (**b**) Schematic of liposome surfaces. The higher surface curvature (smaller diameter) may cause a larger gap between phospholipid head groups than a lower surface

curvature (larger diameter). The larger gap may cause more phospholipid hydrophobic regions to be exposed to the surrounding solution. Scale Bars: a, 3 nm; c, 10 nm.

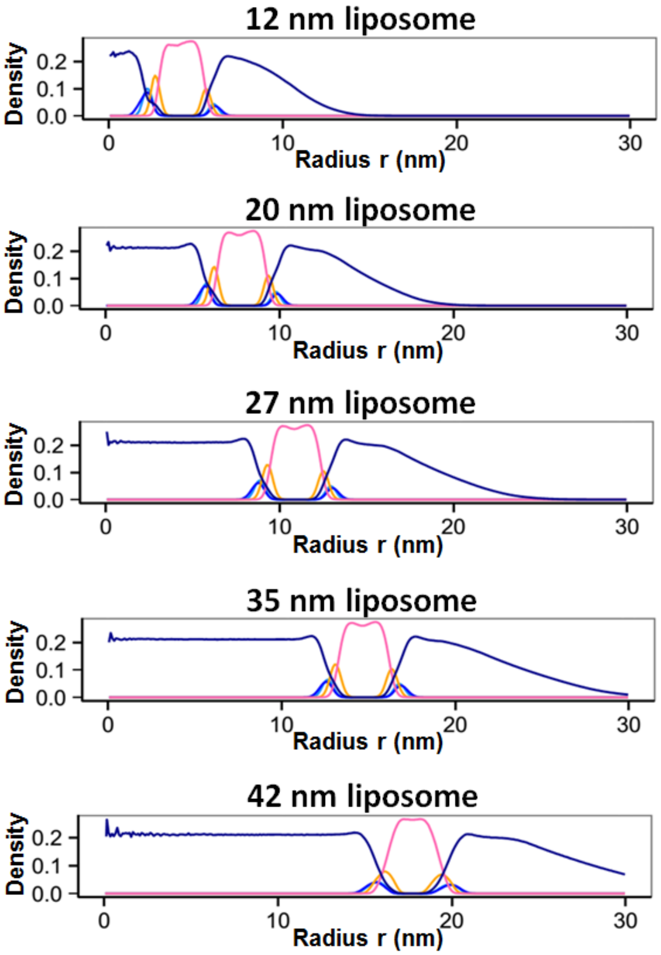

Supplementary Fig. 8: Radial density distribution of components across the liposomes. The density distributions of water (dark blue), PO_4 groups (blue), CHO groups (light blue), glycerol backbone(orange) and lipid tail groups (pink) within each of five energy minimized liposomes were analyzed by comparing radial distribution functions (RDF). The radius was measured from the center of mass of each liposome.

Liposome ID	Number of inner shell lipids (initial/final MD)	Number of outer shell lipids (initial/final MD)	Ratio of outer vs. inner lipid number (initial/final MD)	Number of water molecules	Time to close the pore (ns)	Liposome diameter (nm) (initial/final MD)	Lipid bilayer thickness (r_o-r_i) (nm)
1	157/144	580/593	3.69/4.12	33,898	77	16.2/12.2	6.1-2.2=3.9
2	822/786	1,357/1,393	1.65/1.77	92,530	69	24.2/19.6	9.8-5.8=4.0
3	1,788/1,763	2,578/2,603	1.44/1.48	186,763	152	31.2/26.0	13.0-8.9=4.1
4	3,492/3,415	4,531/4,608	1.30/1.35	352,949	285	39.2 /33.6	16.8-12.7=4.1
5	5,245/5,177	6,521/6,589	1.24/1.27	593,134	494	46.2/39.8	19.9-15.7=4.2

Supplementary Table. 1: Overview of the parameters of five simulated liposomes before and after energy minimization by MD simulation.
